# Why increasing availability of ART is not enough: a rapid, community-based study on how HIV-related stigma impacts engagement to care in rural South Africa

**DOI:** 10.1186/s12889-016-2753-2

**Published:** 2016-01-28

**Authors:** Sarah Treves-Kagan, Wayne T. Steward, Lebogang Ntswane, Robin Haller, Jennifer M. Gilvydis, Harnik Gulati, Scott Barnhart, Sheri A. Lippman

**Affiliations:** 1 University of California, San Francisco, Center for AIDS Prevention Studies, San Francisco, CA USA; 2 University of Washington, International Training and Education Center for Health (ITECH) – South Africa, Pretoria, South Africa; 3 University of California, San Francisco, Global Health Sciences, San Francisco, CA USA; 4 University of Washington, International Training and Education Center for Health, Seattle, WA USA

**Keywords:** HIV, South Africa, Stigma, Engagement to care, Antiretroviral therapy (ART)

## Abstract

**Background:**

Stigma is a known barrier to HIV testing and care. Because access to antiretroviral therapy reduces overt illness and mortality, some scholars theorized that HIV-related stigma would decrease as treatment availability increased. However, the association between ART accessibility and stigma has not been as straightforward as originally predicted.

**Methods:**

We conducted a “situational analysis”—a rapid, community-based qualitative assessment to inform a combination HIV prevention program in high prevalence communities. In the context of this community-based research, we conducted semi-structured interviews and focus groups with 684 individuals in four low-resource sub-districts in North West Province, South Africa. In addition to using this data to inform programming, we examined the impact of stigma on the uptake of services.

**Results:**

Findings suggested that anticipated stigma remains a barrier to care. Although participants reported less enacted stigma, or hostility toward people living with HIV, they also felt that HIV remains synonymous with promiscuity and infidelity. Participants described community members taking steps to avoid being identified as HIV-positive, including avoiding healthcare facilities entirely, using traditional healers, or paying for private doctors. Such behaviors led to delays in testing and accessing care, and problems adhering to medications, especially for men and youth with no other health condition that could plausibly account for their utilization of medical services.

**Conclusions:**

We conclude that providing access to ART alone will not end HIV-related stigma. Instead, individuals will remain hesitant to seek care as long as they fear that doing so will lead to prejudice and discrimination. It is critical to combat this trend by increasing cultural acceptance of being seropositive, integrating HIV care into general primary care and normalizing men and youths’ accessing health care.

## Background

Stigma has been cited as a key driver of the HIV epidemic. As stigma acts as a mechanism to reinforce existing power structures, HIV’s association with debilitation, death, lost capacity to work, and behavior that may be deemed socially ‘deviant’ has allowed HIV-related stigma to be especially pernicious and long-lasting [1–3]. HIV-related stigma has hindered intervention efforts, led to delays in testing, reduced engagement in care, and lowered adherence to treatment and care regimens [3–9]. Increased access to antiretroviral therapy (ART) has greatly reduced the disease’s fatality rate and allowed people living with HIV (PLHIV) to maintain their social and economic roles within their families and communities. Many practitioners, activists, and researchers theorized that the improved access to treatment would subsequently lead to the decline of HIV-related prejudice and discrimination [10–12]. This hypothesis is predicated on people having reasonable access to treatment and on anticipations of stigma not acting as an initial barrier to obtaining treatment. Indeed, the actual association between the introduction of ART and stigma, has not been as straightforward as originally predicted.

Although the rollout of ART was followed by reductions in HIV-related prejudicial attitudes in many African countries [13, 14], recent studies also suggest that stigma persists and continues to be a barrier to HIV treatment and care [15]. Considering stigma’s varied manifestations can help further elucidate these findings. The most readily apparent form, enacted stigma, consists of acts of discrimination perpetrated against someone who has or is perceived to have a scorned condition [16, 17]. Other forms are less obvious because they principally involve individual beliefs. Internalized stigma refers to a person’s own endorsement of prejudicial beliefs about a devalued characteristic [16, 18, 19]. When the person endorsing such beliefs is from the devalued group, internalized stigma effectively becomes a form of self-stigma. By contrast, anticipated stigma refers to beliefs that others hold prejudicial beliefs about a devalued group and that they may discriminate against that group [20]. The introduction of ART would most plausibly affect enacted or internalized stigma. It is possible, however, that people may continue to anticipate the possibility of stigma and take steps to avoid it [15, 16]. Such dynamics would have important implications for how best to address HIV-related stigma in order to increase engagement in HIV care.

We examined community perceptions of stigma and sought to understand if and how these perceptions influence people’s engagement with HIV prevention and treatment within the context of expanding access to ART at four sites in two high prevalence districts in the North West Province of South Africa [21, 22]. The Republic of South Africa, which has the highest number of people living with HIV (6.4 million) and the second highest number of HIV-related deaths in the world, has undertaken mass HIV testing campaigns and decentralized and expanded access to ART [23–25]. South Africa now operates the world’s largest antiretroviral program [26] and has significantly improved access to condoms, HIV testing, and treatment [27]. HIV testing is being integrated into primary care and ART is now available in almost all local health clinics and with broadened eligibility criteria (lifetime treatment for pregnant women, tuberculosis (TB) co-infection and increased qualifying eligibility to CD4 ≤ 500 cells/mm^3^ from previous 350 cells/mm^3^). However, huge gaps in testing and treatment remain. Data from 2012 indicate that only 37.8 % of HIV-positive men and 55 % of HIV positive women were aware of their HIV status [23]. Nationally it is estimated that only half of ART eligible patients are currently on treatment [28]. It is thus imperative to understand why people are not linking to or remaining in care and what role stigma continues to play regarding prevention and treatment.

## Methods

We conducted a secondary data analysis on data collected as part of the rapid community-based qualitative assessment to inform a combination HIV prevention project being conducted in the Bojanala Platinum and Dr. Ruth Segomotsi Mompati Districts of South Africa [21, 22]. The primary purpose of the research was to engage community members and stakeholders and tailor intervention activities to the local context prior to program implementation. To do so, a situational analysis was conducted. Situational analysis is a type of rapid assessment that provides a snapshot of the epidemic in context, and includes a review of available data, exploration of new data, and triangulation of the varied data sources, relying heavily on interpretive help of local stakeholders. Further the methods’ data collection and analysis balances gathering a breadth and depth of data while adhering to a short timeframe. In today’s context of diminishing resources for health programming, rapid assessments provide an efficient means to improve the efficacy and acceptability of targeted HIV prevention programs [29, 30]. One week of rapid data collection, including in-depth-interviews (IDIs), focus group discussions (FGDs), and health facility assessments, was conducted in each site between April 2012 and September 2013. Details on methods and results, including impact on program design, are described elsewhere [21, 22].

### Study sites

The situational analysis was undertaken in four sub-districts in North West Province, which has the fourth highest HIV prevalence in the country (estimated at 30.2 % in the antenatal setting [31]). Study sub-districts were selected for inclusion by South Africa Department of Health collaborators. Generally, the sites are characterized by poverty, unemployment, and low literacy levels, however industry and population density in the study areas varied from the urban Rustenburg sub-district, home to the platinum mining industry and rapid population growth, to Lekwa Teemane sub-district, a compact rural area dominated by farming.

### Data collection team

Data was collected by a team of up to 11 trained clinicians, social scientists, epidemiologists, program staff, and government officials. Training included ethics, confidentiality and consent procedures; orientation to study goals and data collection instruments; interviewing, facilitating, and note taking skills; documentation and data management procedures; and analysis skills. Team members who were new to qualitative research were paired with more experienced researchers for the first few days of fieldwork.

### Recruitment & eligibility

Following stakeholder meetings in each sub-district, the data collection team spent one week at each site visiting health and social service locations, non-governmental organizations (NGOs), and areas where vulnerable and mobile populations congregate, such as truck stops, taxi ranks, taverns, and informal settlements. Facility assessments were conducted at all health facilities in the two smaller sub-districts and at a purposefully sampled, diverse subset of health facilities in the two larger sub-districts. For interviews and focus groups, teams spoke with key informants (e.g. government officials, employers, and community leaders), health care providers, and community members (Table 1). Participants were selected through a combination of convenience and purposeful sampling, based on stakeholder recommendations. People were eligible to participate if they were age 18 years or older, spent time in the respective sub-district, were not under the influence of drugs or alcohol, and were able and willing to consent. Written informed consent preceded all interviews; consent and interviews were conducted in English, IsiXhosa, IsiZulu or Setswana, based on participant preference. All procedures were approved by the Committee for Human Research at the University of California, San Francisco (UCSF), and the Human Sciences Research Council (HSRC) Research Ethics Committee in South Africa. As this data collection was performed to inform the development of a non-research intervention, the Human Subjects Division at University of Washington and CDC’s Center for Global Health Human Research Protection determined that the study did not meet the regulatory definition of research under 45 CFR 46.102(d).

**Table 1:** Number of participants in interviews, focus groups and assessments, by place and type

		Naledi	Moses Kotane	Lekwa Teemane	Rustenburg	Total
*Type*	*Description*	*Number of participants*				
Provider interview	Nurses, facility managers, doctors, pharmacists, HIV counselors, social workers, lab technicians and data capturers.	33	26	21	18	98
Key informant interview	Farm (Naledi) and mine management (Moses Kotane), social development workers, non-governmental organizations (NGOs), home based carers, traditional healers, traditional leaders, ward councillors, Department of Health (DOH) officials, religious leaders and tavern owners.	38	22	37	24	121
Community member interview	Young adults (18–35), sex workers, mobile workforce (e.g. truck drivers), migrant populations, people living with HIV, church members, tavern clients, farm workers (Naledi, Lekwa Teemane), mine workers (Moses Kotane, Rustenburg), people living in informal settlements, men who have sex with men, self-identified lesbian, gay, bisexual, or transgender individuals.	57	32	68	57	214
Focus group	Primarily community members sharing a common characteristic (occupation, youth, religion, etc.); also included some key informants such as home based care workers and NGO staff.	63	70	50	68	251
		*Number of facilities*				
Facility assessment	Clinics, community health centers, hospitals, roadside clinics and mobile clinics.	4^a^	8	7^a^	8	27

^a^represents all clinics in the sub-districts, exclusive of hospitals

### Interviews, focus groups and facility assessments

Semi-structured interview guides included questions regarding community characteristics, mobility, employment, health challenges, TB and HIV-related services, stigma related to HIV or TB, sexual partnerships, and suggestions for health programming in the area. Each guide had a specific section on HIV-related stigma; example questions included “What kinds of challenges do people living with HIV face in this community?” and “If you were HIV positive, would you talk about it with other people? Why/Why not?” No participant was asked their HIV status, although many voluntarily shared it. As we did not systematically collect data on participant’s HIV status, we did not formally include participant HIV status in our analysis. Health facility assessments were conducted to document local health resources and gaps in services. For the present analysis, we utilized facility assessment variables that emerged as being directly relevant to exacerbating (or not) stigmatization of people living with HIV (PLHIV) in the clinic setting, such as confidentiality procedures, infrastructure, auditory and visual privacy, sufficient staffing levels, and data management systems.

The study team took detailed field notes of each interaction and recorded interviews when participants consented to be recorded. All field notes were recorded in English, regardless of the language of the interview or focus group, with the study team simultaneously translating for their field notebook as needed. Facility assessment data was recorded on a standardized form with room for researcher notes and observations.

### Data analysis

Our data analysis process included two main phases of analysis. The first phase was an iterative analytic process undertaken in the field by the entire data collection team (conducted by STK, LN, RH, SAL, JMG) [21, 32]. The entire fieldwork team met at the end of each data collection day to discuss findings, pinpoint unanswered questions or knowledge gaps, offer new leads, and prioritize subsequent interviews. By the end of the week, the research team had agreed on the principal themes that were emerging from the data, on key venues and areas to focus prevention efforts, and on key questions that required further investigation [21]. These principal themes formed our initial analytical and coding framework.

The second phase of data analysis commenced upon returning from the field. This subsequent analysis (conducted by STK, RH, HG) consisted of reviewing meeting notes and coding original textual data from the fieldwork notebooks. For the present analysis, we utilized the codes of stigma, HIV-related concern and awareness, condom use, testing, disclosure, treatment, barriers to accessing care, and communication. All interview notebooks were coded by hand (STK) and recordings were referenced whenever questions arose based on the notes or as quotes were needed. As the main purpose of data collection was program planning and a significant proportion of the analysis was conducted while in the field, only a small number (*n* = 31) of interviews and focus groups were transcribed for analysis in ATLAS.ti (ATLAS.ti Scientific Software Development GmbH, Berlin, Germany). This included interviews with 13 key informants, 10 providers, four community members, and four FGDs (with 25 community member participants). Interviews were chosen for transcription purposefully to ensure inclusion of key information from informants when language of interview made it difficult to cull important themes from mixed language notes (as all handwritten notes were analyzed) and for under-represented groups in the data.

Code reports were generated and reviewed. Based on review of the deductively coded data (both transcripts and notes), the authors (STK, WTS, SAL) developed inductive codes to organize data on themes that emerged within transcripts and interview notes and across the topics. All interview notes were re-read to cross-check findings and to ensure representation from all data collected. Authors (STK, WTS, SAL) discussed and clarified the main ideas that emerged and explored relationships within the data among themes [33, 34]. Data from facility assessments were summarized and compared to findings from the interviews. We interpret and present the data in the aggregate because the major themes did not differ across sub-districts or sub-populations, except where specifically noted.

## Results

Across the four sites, 684 participants, including key informants, providers, and community members, participated in in-depth interviews (433) and focus groups (251). Twenty-seven facility assessments were conducted (Table 1). A little more than half of the participants were female. Specific data on other identifiers, such as age or HIV status, were not recorded.

In the sections that follow, we describe our findings on the manifestations of stigma, how people attempt to avoid disclosure, the resulting impact on engagement in care, and strategies to overcome stigma’s effects. Table 2 provides a brief overview and examples from our results.

**Table 2:** Disclosure risks, impacts to engagement in care and mechanisms to avoid disclosure

**The impact of increased availability of ART**
Fewer HIV-related deaths reported, HIV has transitioned from a “death sentence” to a chronic disease
HIV-related stigma declining but still present
HIV remains highly associated with promiscuity and adultery
**Efforts to control knowledge about a person’s HIV infection**
Avoided disclosure for fear of abandonment or prejudice
“Counterfeiting,” or citing TB, other illnesses or witchcraft as cause of illness instead of HIV, a common way to avoid disclosure
Did not take treatment to avoid explaining need for medications to family or people they are living with
**Seeking care is in conflict with keeping HIV status private**
Being seen at the clinic (for any reason) caused suspicion of HIV or gossiping; this significantly delayed HIV testing or engagement in care and was especially problematic for youth and men
Home based care workers visiting a house could signal to neighbors that someone was HIV positive; false contact information given or care from home based care workers was refused
Clinic infrastructure such as HIV specific rooms, filing systems, different colored folders and coding systems revealed HIV status to other patients
There was a severe distrust of health care workers breaking confidentiality, partially fuelled by patients knowing nurses at local health facilities
**Attempts to increase engagement to care and combat stigma met with varying success**
Reduced initiation of treatment or adherence because treatment had to be picked up at clinics.
Community members spend more money and/or time to go to a private doctor or attend facilities in a different community
Clinics tried to facilitate support groups or encourage an ART “supporter” for PLHIV—these were met with varying success
Male dominated spaces (i.e. mine health facilities & truck stop clinics) were more successful in engaging men in care

### The impact of increased availability of ART

As respondents reflected back on the course of the HIV epidemic in their community, they reported that HIV-related deaths were much less common than in the past and reported increasing acceptance of HIV. Many participants used similar language to describe HIV’s transition from a *“death sentence”* to a “*chronic disease, just like diabetes*” that could be managed now that treatment is available.

*“I think the scourge of HIV and AIDS is the one that's -- it has had a terrible impact in the past. It's now quieter…" [#1, key informant, male, site #1]*

*“Like myself, everyone was afraid. Before it was a life sentence, you were going to die. Everyone was panicking—it was horrible. Now, if you know your status you are going to get help.” [#2, key informant, female, site #4]*

Providers, people living with HIV who were taking part in support groups, those who worked with NGOs delivering HIV-related services, and those in the urban areas were most likely to regard HIV-related enacted stigma as declining. Community members also reported declining discrimination, with most narratives describing improvement, but not resolution, in how individuals with HIV infection were treated.

*“Everybody is starting to accept HIV. Even though stigma is still there, it’s very little. It’s not like before…It’s not that they would hate you…but it’s like ‘I don’t want to be with this person as my partner, who is HIV positive.’” [#3, community member, male, site #4]*

HIV remained highly associated with promiscuity and adultery, however, and prejudicial beliefs were still reported:

*“[A]nd of course, people always look at [HIV] as a moral issue. That's why you find someone who -- you know, who is infected would feel they should hide it, because when people talk about this, they would always be saying it's people that behave in this fashion, or that fashion. Like people that are promiscuous and all of that.” [#4, key informant, male, site #1]*

Informants spoke at length about continuing negative social consequences of living with HIV. They noted that, even though enacted stigma may be less severe than before, HIV positive community members could still be treated differently or socially isolated, both from the general community and from family.

*“They just shun you. Today they like you, but stigma is a problem because sometimes if somebody finds out I’m HIV positive, the treatment will never be the same again.... tomorrow you're not one of them.” [#5, key informant, female, site #1]*

*“Now he thinks that person doesn’t have to eat with my spoon, doesn’t have to wash my dishes. Doesn’t have to sleep with us, he have to sleep alone. That is making a stigma, and that’s why I think most of the people they died because of that.” [#6, focus group member, female, site #1]*

Participants also reported feeling stigmatized by healthcare providers. This was especially common among key populations: youth, sex workers, and men who have sex with men (MSM). Healthcare workers themselves were impacted by concerns of facing stigmatization while receiving care, with some key informants and facility managers reporting that healthcare workers didn’t seek care for fear of their colleagues’ reactions. *“It’s sad that some [clinic] staff will die due to lack of being monitored. This is a serious challenge.” [#7, key informant, female, site #4]*

### Efforts to control knowledge about a person's HIV infection

Though the general consensus was that HIV enacted stigma was not as extreme as it once had been, participants universally reported that most HIV-positive men and women did not disclose their HIV status for fear of social ostracism (i.e., anticipated stigma). Participants reported that disclosure to partners could lead to breaking up or abandonment, blame, accusations, and violence. Participants reported fears that family members might ostracize individuals who revealed their HIV status or refuse to accept that the person is HIV-positive.

*“If I was positive, I would never disclose. People attack your background; you don’t get support” [#8, key informant, female, site #3]*

*“I can say that [their HIV status] is a person's secret because eh…sometimes when other people know they ridicule you or on and on. Now that is why it is a secret… because it is said even if the doctor knows it becomes yours and the doctor's [secret]…there is no need to tell anybody…” [#9, community member, male, site #2]*

Participants consistently reported that PLHIV use strategies to manage who learns of their HIV status. Many participants reported PLHIV prefer to “counterfeit” [35, 36] or pass off their HIV infection as being something else to avoid disclosing their HIV status. For example, one participant was interviewed at a heath facility where he had gone to collect his ART refill. He had told his family that he was going to the clinic to get treatment for a recent car accident. More often, TB was used as the alternate explanation for their health problems, although it too is stigmatized to a lesser degree.

*“People know that they died of HIV but they shield it – TB, TB, TB” [#10, key informant, female, site #3]*

*“People who had TB in the past never used to disclose it because of the stigma. But it has shifted to HIV. So everybody who is HIV positive, they won’t say I’m HIV, they will say that I’ve got TB. So TB’s a better disease than HIV because they know TB can be cured. But HIV cannot.” [#11, key informant, female, site #1]*

Participants also turned to the common belief in bewitchment or “makgome,” a disease without cause, and the use of traditional healers as a means of concealing their HIV infection and avoiding the clinic:

*“This person will know that ‘I’m HIV positive’. But already everybody in the family thinks its makgome. So it is very difficult for this person to disclose. Because how is he or she going to tell them that, ‘I’m HIV positive’?” … Its denial but also coping.” [#11, key informant, female, site #1]*

Participants relayed significant adherence challenges due to reluctance to disclose to partners, family or friends. It was common to have many people living in the same house, which complicated efforts to take medications discreetly. One participant spoke of an HIV-positive man who came regularly to collect his medications and even participated in pill counts to ensure adherence. When he died, his family members, to whom he had not disclosed, found all of his pills underneath his bed. A lack of family support and sense of isolation further decreased people’s abilities and desires to seek care or adhere to treatment.

*“Lots of people are dying because they can’t go out and talk.” [#12, community member, female, site #3]*

### Seeking care is in conflict with keeping HIV status private

Participants reported that simply being *seen* at a health clinic meant risking exposure of one’s HIV status. This perception was considered to be a major barrier to accessing health facilities for testing or treatment and a reason for delaying access to care until extremely sick.

*“Cuz they think…you will see him go to the clinic and maybe you will think that, ‘Oh, he has the HIV and AIDS’.” [#13, community member, female, site #1]*

*“I still believe that there is the issue of stigmatization, we see a major problem because we find that that…some of these people come [to clinic] when they are in the last stage [of disease].” [#14, provider, male, site #2]*

This barrier manifested itself differently for men and women. Participants reported that men were much more likely to wait to seek care until they were very sick. This difference was due in part to local notions of masculinity, which disfavor seeking help because it is thought to be a sign of weakness.

Focus Group Member 1 (male): *“I’ll refer to boys specifically. It seems like their dignity is going to fall being seen entering a clinic or something…or a hospital.”*

Focus Group Member 2 (female): *“There is the concept that the clinic is only for females.”*

Focus Group Member 1: *“[This idea] has passed on from one generation to the other.” [#15, focus group for youth, site #3]*

Men felt at risk of being stigmatized because being sick and attending a clinic “for females” was considered a deviation from the ideal notions of masculinity. The gender difference was also tied to broader variations in why men or women would be seeking services at a clinic in the first place. Women have an easier time accessing regular care for HIV because they can do so under the guise of reproductive health services, pregnancy, or children’s health care needs. Men and youth, by contrast, do not have the same clinical reasons to be seen at a health care facility, and their attendance could lead to gossip. For youth, the situation was further complicated by fear that someone would tell their parents they were at a facility.

*“But to see people who go out of their free will [to test for HIV], especially males, I find it rare. I think with males…my assumption is that people think they are healthy or the man is worried to find out [his HIV status], you see. But you know with women, when you're pregnant you go, you test. When they go for their prevention contraceptives, they are tested. With females I find they have the tendency of knowing they can go and test any time.” [#16, key informant, male, site #1]*

Providers reported that fear of stigmatization also led patients to give incorrect contact information to prevent the health facility from contacting them for treatment or additional tests. Respondents reported that family members sometimes prevented home-based care workers from visiting sick relatives because they did not want neighbors to know that someone in the house had HIV, as home based care workers wore uniforms and were therefore easily identifiable. A home based care worker reported:

*“Maybe it’s few of them that ignore it and they’re scared to tell other people, even me, the [caregiver], and they can’t talk to me and say, ‘Hey, sister. I’ve got this. What can I do? And I need help.’ They ignore it and at the end of the day, is dying.” [#6, focus group member, female, site #1]*

Infrastructure also played a role in keeping PLHIV from accessing care due to fears of disclosure. Informants reported, and study staff observed, extremely full facilities with long wait times and physical infrastructure not designed to serve such a high patient load. As with many rural areas, recruiting and maintaining staff was also a significant challenge [37]. Staffing levels were reported to be insufficient to meet the needs of the local population at 22 of the 27 (81 %) health facilities assessed. As a result, some clinics attempted to see patients more efficiently by instituting separate waiting areas and consulting rooms for HIV testing to streamline the care process. Some facilities also implemented specific days of the week for specific health issues, e.g. Thursdays were for patients with “chronic” conditions, which includes HIV-positive patients. This euphemism was not lost on the community. One community-member explained that a separate building was used at one clinic to dispense ART to HIV positive patients:

*“[That building] is in the same yard [as the clinic] so obviously people see you going there [to collect treatment] and that causes defaulting [i.e., not adhering to ART medication regimens].” [#17, community member, female, site #4]*

Further, data management practices lacked uniformity and easily revealed the HIV status of patients. Of the 14 facilities where the field team specifically compared medical record keeping practices for HIV positive versus other patients, 11 (73 %) used different file labels, folder colors, or storage systems for HIV patient files. Although these practices were instituted for non-stigmatizing reasons, such as a lack of space or a desire for greater efficiency, they were seen by participants as a potential means by which a patient’s confidentiality could be unintentionally violated and his/her HIV status disclosed.

Participants also reported that many community members were reluctant to be tested for HIV or receive HIV care at local clinics out of fear that healthcare staff would reveal their status. Some participants could not point to specific examples of confidentiality breaches, but still described wariness of clinical environments. Others were able to cite specific examples of breaches, with a handful of key informants even reporting that they had misused their own access to health records to look up the status of someone they knew to be sick. One key informant reported:

*“Recently someone (a health care provider)…was drunk…and said that our village is full of people who are HIV-positive and that [s/he] could even expose them by their names. This person even mentioned another family by name. As a result many are scared to get their treatment… because they do not want their names spread all over the place.” [#18, key informant, female, site #2]*

Fear of health worker gossip was particularly keen in rural areas and smaller towns—“*In a small town, everyone knows everything.” [#19, key informant, female, site #3]*

Facility assessment data confirmed insufficient attention to confidentiality. Of the 26 facilities accessed, 14 (54 %) reported not having a confidentiality policy; 6 (23 %) of the facilities produced a written confidentiality policy for the field staff; and the remaining 6 (23 %) reported having a policy but couldn’t not produce the materials to the field staff.

*“What they tend to say is that I don't want to test at the clinic because I know the counselor and if she finds out I’m positive she'll probably go tell my friends. …So that's why you'd find that some of the people that do test don't even test in the community.” [#5, key informant, female, site #1]*

### Attempts to increase engagement to care and combat stigma met with varying success

The perceived risks of being seen at the clinic, having HIV status effectively disclosed through clinic practices (e.g., HIV-specific waiting rooms), or gossip by healthcare workers led many patients to either not seek services or to receive them in distant communities, despite the fact that transportation to and from distant facilities was a significant challenge. In areas where private health care was available, participants also reported spending money to receive treatment in a more confidential setting instead of utilizing the free care at the public health facilities.

Providers in some areas have launched efforts to increase engagement in care, for example, by establishing support groups. Participants in those groups reported beneficial effects. *“It kills you if you keep it in your heart. It will hurt you. It’s better to share these things together.” [#20, community member, female, site #1]* However, informants also noted that the support groups were of limited impact and hard to sustain because many people were afraid of being stigmatized if they participated. Other health facilities strongly encouraged HIV-positive patients to bring a “supporter”—a friend or family member who will help them adhere to treatment—to the clinic at the time of ART initiation. Some participants felt this practice dissuaded those who are not ready to disclose from returning to the clinic or beginning treatment.

Some efforts were designed specifically to increase the proportion of men in care. For example, a couple of areas had small clinics that operated at night at truck stops and employed ‘peer educators’ to recruit truck drivers. Although the subset of patients reached was modest, it consisted almost entirely of men. In addition, at some large mines, employees were required to complete health screenings when first hired and when returning from long leaves, a practice that gave men a reason for being seen at a healthcare facility. HIV testing was offered during this process.

## Discussion

We found that increased accessibility of ART had a positive impact in the community and that reports of enacted stigma were on the decline, but still present. In contrast to theories predicting that stigma would dissipate once PLHIV had access to effective treatment (because HIV would no longer be associated with death and lost economic productivity) [9–12], community perceptions of *anticipated* stigma persisted. This persistence meant that the current, dominant primary outcome of stigma was PLHIV hiding their HIV status from others. This desire to conceal HIV status, or to manage anticipated stigma, was, in turn, a hindrance to engaging in care in this high prevalence, rural setting. These findings help to refine our understanding of the role of stigma in a country where improvements to ART access are ongoing, and to explain differences observed in other research. Prior study results were mixed on the continuing impact of stigma. For example, stigma has been shown to be a major barrier to initiating treatment [38]. But after initiation, stigma has no impact on whether clients discontinue their medications [39]. Instead, ART initiation leads to increased support [9]. Our data explain why all of of these things can be simultaneously true. As reflected in the findings, a major impact of stigma comes from what a person fears might happen, which in turn dissuades people from seeking treatment. Once engaged in care, however, the impact of stigma is less straightforward and likely to be dependent on the specific context in which a person accesses care and the reactions a person receives from others. Given that community members’ hostile attitudes toward HIV have lessened, some people living with HIV may find that prejudice and discrimination are not as big of problems as they had feared. As a result, these individuals are then able to remain engaged in care and to identify support among friends and family.

The shift in the dominant manifestation of stigma (from enacted to anticipated) is in line with predictions from classic stigma theory [40]. Access to ART reduces overt signs of HIV infection, effectively changing the disease from a discredited (readily apparent) to a discreditable (hidden) stigmatizing condition [40]. As a result, PLHIV are now less likely to encounter enacted discrimination because they pass as HIV-uninfected in many social situations. Furthermore, the declines in enacted stigma are likely reinforced by the continuing high prevalence of anticipated stigma. As reflected in our participants’ reports, HIV-positive individuals fear that others will be prejudiced against them. In turn, they take steps to limit who knows of their infection, which then further reduces the likelihood of encountering discriminatory behaviors [16, 41–43]. Previous qualitative work in South Africa has similarly found that having fewer physical symptoms due to treatment helped reduce some stigma, but that fear of being discovered to be HIV positive remained [44]. Unfortunately, the efforts to conceal one’s HIV status can have serious consequences. Most immediately, a person is able to avoid discrimination [16, 45], an outcome that desirable and likely reinforcing. But over the longer-term, there are potential deleterious mental and physical health impacts. People who chronically hide their HIV-positive status are more likely to experience depression, cut off access to valuable support resources, and potentially delay needed care [16, 45, 46].

Our findings contribute to a complex field of study and serve to explain seemingly divergent trends. Some studies in Sub-Saharan Africa have found that ART availability has ‘normalized’ HIV [12–14] while others have found increases in anticipated stigma despite increased availability of ART [15, 47]. We found that, in fact, both realities exist. HIV is stigmatized both because it is a potentially deadly infectious disease and because it is associated with behaviors traditionally considered shameful [1]. The introduction of ART has altered the deadliness of the disease, thereby lessening one driver of stigma and removing the motivation for some of the most extreme forms of discrimination (e.g., avoiding even minimal contact with people living with HIV). It has not, however, changed the disease’s association with scorned behavior, which means that prejudicial attitudes and discriminatory behaviors are still possible. People with HIV have valid reasons to remain wary of others learning of their infection. This reality reflects stigma’s continuing role in perpetuating existing power structures and social norms, such as placing restrictions over women’s sexuality and reinforcing masculine norms that idealize being “healthy and virile” and not needing help or health care [3, 22].

It is important to note that our data collection spanned over a year and a half, leading to differences in how long enhanced access to ART had been available in different sub-districts. These differences were due both to how much time had elapsed since the enhanced ART program officially launched and to the process by which changes were disseminated. In our study sites, municipals hubs and semi-urban areas tend to implement health system changes more quickly compared to expansive, rural sites. It is possible that as more time passes in which ART is available, the relationship between engagement in care and HIV-related stigma may further evolve. However, without addressing why HIV is stigmatized and without changing the context in which HIV care is accessed (which currently can lead to unintended disclosure and discrimination), we would expect the relationship to stay the same.

### Implications for HIV prevention and treatment programming

These results have informed a large-scale comprehensive HIV prevention program to address social barriers to prevention and care. Four comprehensive prevention strategies that were implemented following the situational analysis include community-based comprehensive health and HIV testing campaigns; support groups for people living with HIV; health systems strengthening, including a focus on de-stigmatizing HIV and HIV care; and community engagement, including training modules for stigma reduction with key stakeholders. Below we suggest mechanisms to both reduce stigma and/or to allow people to access care even in spite of stigma, some of which we were able to incorporate into our current programming.

Given the interpersonal and structural dynamics affecting stigma and HIV testing and treatment, interventions that address different facets of the problem are needed. First and foremost, stigma reduction programming must operate in tandem with the rollout of ART [48]. In fact, South Africa has already launched a national stigma reduction campaign and is moving forward with significant improvements to its HIV care systems while expanding access for early treatment. Outreach and training programs have been introduced for health care facilities; however, programming that includes discussion around stigma could be extended beyond health care workers into the general community to help orient family members and friends to providing support for loved ones who are HIV-positive. Second, those living with HIV should be provided with tools to cope with the discrimination and hostile attitudes that they may encounter in daily life, as well as to address internalized feelings of shame [49]. This can be done through establishing support groups for people living with HIV/AIDS (PLHIV) to encourage treatment adherence and to create an environment conducive for building social networks that can reduce stigma and engender support. Although this second approach does not necessarily address the larger structural problems of stigma in the community, it is an important stopgap measure to ensure that people living with HIV have the needed skills to enhance their engagement in care and maximize both HIV and mental health outcomes.

Third, structural shifts in the resources to manage ART distribution could also help to reduce stigma, along with enhancing access to care. Currently, the impact of stigma anticipations on treatment access is exacerbated by health system capacity problems. Clinics are not able to manage their patient loads efficiently and confidentially because they lack sufficient space or trainings in confidentiality-related best practices*.* Participants in our study reported lack of privacy, data management processes that effectively revealed their HIV status, and lack of trust in providers maintaining patient confidentiality, which resulted in delayed testing and treatment and a greater risk of falling out of care. This violates both South African and universal best practices and standards of care to which all patients, regardless of HIV status, are entitled.

The National Department of Health is currently encouraging facilities to have an “integrated” care model (i.e. not having separate spaces for HIV testing, etc.) and rolling out an electronic data management system—which in the study sub-districts was largely still paper-based at the time of data collection. When completed, both transitions may eliminate some current practices that could reveal patients’ HIV status. Modifying existing infrastructure to accommodate visual and auditory privacy as more patients seek testing and qualify for care is paramount. Strict policies should be implemented to secure patient health information [50], along with prioritizing staff training on confidentiality, sensitivity and stigma reduction. Concerted efforts to rebuild trust between health facilities and the community could improve access to care [51]. Other studies on stigma in Sub-Saharan Africa and South Africa have also found that HIV-related stigma, fear of rejection and stigmatization, lack of confidence in confidentiality protections and provider mistrust result in refusing or delaying HIV counseling and testing services [52–54]. Stigma specifically in healthcare settings has also been found to be associated with low access to care and poorer physical and mental health outcomes [53, 55, 56]. Addressing these problems is a critical component of an overall effort to reduce HIV stigma.

Finally addressing and responding to social norms around how health care is accessed can serve to increase engagement to care. In line with previous research in South Africa, we found that HIV stigma intersects with prevailing norms around healthcare utilization and masculinity. Participants reported and the research team observed that the overwhelming majority of patients at health facilities were women, children, and the elderly. This trend was facilitated by the fact that these individuals commonly have non-HIV, non-scorned reasons to be at a clinic. In a culture where public health is conceptualized around women and children, men would have few health care needs outside of illness. HIV, which heavily impacts men at an age when they are not likely to have other chronic conditions, becomes the default condition associated with men's clinic attendance. Previous research in South Africa found that men who recently initiated ART felt that seeking care put them at risk for being labeled as HIV-positive and that men view public health facilities as women’s spaces [57–59]. To avoid being labeled as HIV-positive, participants noted that many men delay care or minimize interaction with clinics. Traditional ideas of masculinity further exacerbate the relationship between stigma, engagement to care and gender norms. We found that clinic attendance and illness are interpreted to be signs of weakness and deviations from the ideal of men as healthy and virile. Previous research findings from South Africa have also documented this relationship, and showed that straying from the traditional ideals of masculinity leads to increased anticipations of stigma and undermines health-seeking behavior [60–62].

Improving men’s engagement to care in South Africa is critical—research shows that men test and enter care later, present with lower CD4 counts, and have worse HIV-related outcomes than women [63–66]. Re-envisioning men’s health and reframing conceptualizations of why and how patients seek care will benefit HIV-related care and men’s overall wellbeing [67]. We created community workshops regarding gender (in addition to the community workshops on stigma reduction) to engage with men and women to challenge traditional ideas of masculinity regarding health. This type of programming has already been shown to increase HIV testing [68–70], and has the potential to increase engagement in care [71]. Men and youth need to be able to seek care without raising suspicions that they are HIV-positive. As described earlier, men at health facilities near mines were better able to engage in HIV care as it was delivered as part of work-related health services, leading to less risk of revealing a person’s status. Indeed, we designed a portion of our community testing campaigns specifically to target men—holding confidential testing events at local mines. Programming for men’s wellness is a promising approach that merits exploration.

### Limitations

The data utilized in this analysis were drawn from a larger situational analysis intended to broadly understand the local HIV-related healthcare systems. Although stigma was not the primary focus of the project at the outset, it emerged in the course of data collection as a key theme [22]. As a result, it is possible that our interviews and analysis may have missed nuances in how stigma manifests, as we did not purposefully target HIV-positive participants for interviews (though many community members revealed their status in the course of study participation). Because of our approach, many of the interviews focused more generally on how individuals in the community responded to stigma, rather than first person accounts of its effects on a participant’s own life. Furthermore, our use of focus groups with subset of participants may have reduced individual’s willingness to disclose sensitive information. Nonetheless, we believe it is unlikely to have influenced our findings. There was strong concordance among what different participants reported (e.g., patients feared that providers might judge them for having HIV, some clinic staff reported using access to confidential information to figure out if a person has HIV). There was also strong concordance between the information that participants reported and what our study staff directly observed in the process of collecting data (e.g., participants feared that people may learn of their HIV status if seeking care, study staff observed clinic procedures that effectively revealed who is seeking care for HIV).

Further, our methodology is a rapid, community based methodology. Inherent to rapid methodologies, there was limited opportunity to engage with every population group in-depth and limited resources to transcribe all interviews. In general, there is always a balance of rigor with prolonged timelines and additional expense. This process allowed for extensive qualitative data collection and analysis over a short period of time. It is also possible that our purposeful and convenience-based sampling methodology or only transcribing a small percentage of our interviews introduced bias into our results. However, the large amount of data collected, the breadth of data collection targets and the multi-stage analysis plan, including analysis in the field and reading all handwritten notes twice, buffers against extreme bias.

## Conclusions

Although significant strides have been made in creating stigma reduction interventions [72], HIV stigma continues to be a major barrier to reducing HIV incidence and mortality [53, 73]. As such, reducing stigma, motivating community members to know their status and engage in care, and providing tools for individuals and families to support people living with HIV continue to be foundational to HIV prevention and care work [74–76]. Prevention and treatment programming can be made more effective by identifying how stigma hinders engagement to care and implementing interventions that specifically address those factors [77]. Further, efforts to find opportunities to increase access to health care, in spite of stigma, such as ensuring patient confidentiality and as creating safe spaces for men and youth to access care, cannot be ignored and merit further exploration.

## Abbreviations

ART: anti-retroviral treatment
FGD: focus group discussion
HIV: human immunodeficiency virus
IDI: In-depth interview
MSM: men who have sex with men
NGO: non-governmental organizations
PLHIV: people living with HIV
